# Supplementary material for: Comparative genomics provides new insights into the diversity, physiology, and sexuality of the only industrially exploited tremellomycete: Phaffia rhodozyma
Source: BMC Genomics. 2016 Nov 9;17:901. doi: 10.1186/s12864-016-3244-7 (PMC5103461; doi:10.1186/s12864-016-3244-7)
Supplement: Additional file 6: — List of orphan genes with links to PFAM (related to Additional file 1: Table S1). (ZIP 1428 kb) [file 12864_2016_3244_MOESM6_ESM.zip › PFAM_UNIQUE/G04110_P.html]

#                                                                            --- full sequence --- -------------- this domain -------------   hmm coord   ali coord   env coord
  
# target name        accession   tlen query name           accession   qlen   E-value  score  bias   #  of  c-Evalue  i-Evalue  score  bias  from    to  from    to  from    to  acc description of target
  
#------------------- ---------- ----- -------------------- ---------- ----- --------- ------ ----- --- --- --------- --------- ------ ----- ----- ----- ----- ----- ----- ----- ---- ---------------------
  
G04110\_P             -            851 Phostensin\_N         PF13916.1     82      0.58    8.9   8.1   1   2   0.00077       2.3    6.9   0.4    11    42   470   504   463   516 0.74 - PP1-regulatory protein, Phostensin N-terminal  
G04110\_P             -            851 Phostensin\_N         PF13916.1     82      0.58    8.9   8.1   2   2    0.0074        22    3.8   2.2    56    72   573   589   558   594 0.78 - PP1-regulatory protein, Phostensin N-terminal
